# Supplementary figures and images for: Arousal state affects perceptual decision-making by modulating hierarchical sensory processing in a large-scale visual system model
Source: PLoS Comput Biol. 2022 Apr 4;18(4):e1009976. doi: 10.1371/journal.pcbi.1009976 (PMC9009767; doi:10.1371/journal.pcbi.1009976)

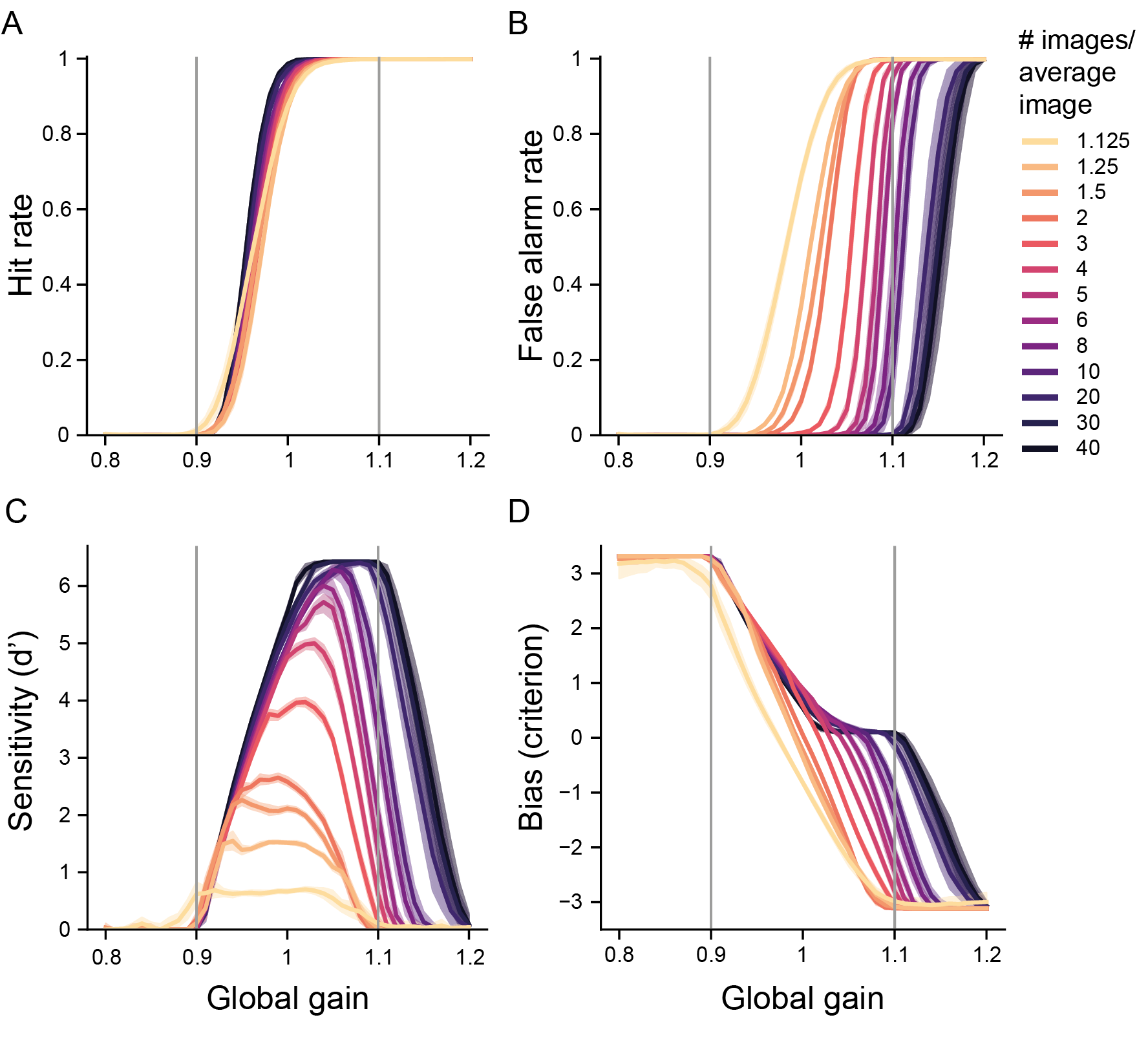

Supplement: S1 Fig — Shaded areas depict the 95% confidence interval across ten model instances. The grey vertical lines are in reference to Fig 2D and serve to illustrate the results for a more narrowly sampled global gain range. (TIF) [file pcbi.1009976.s001.tif]

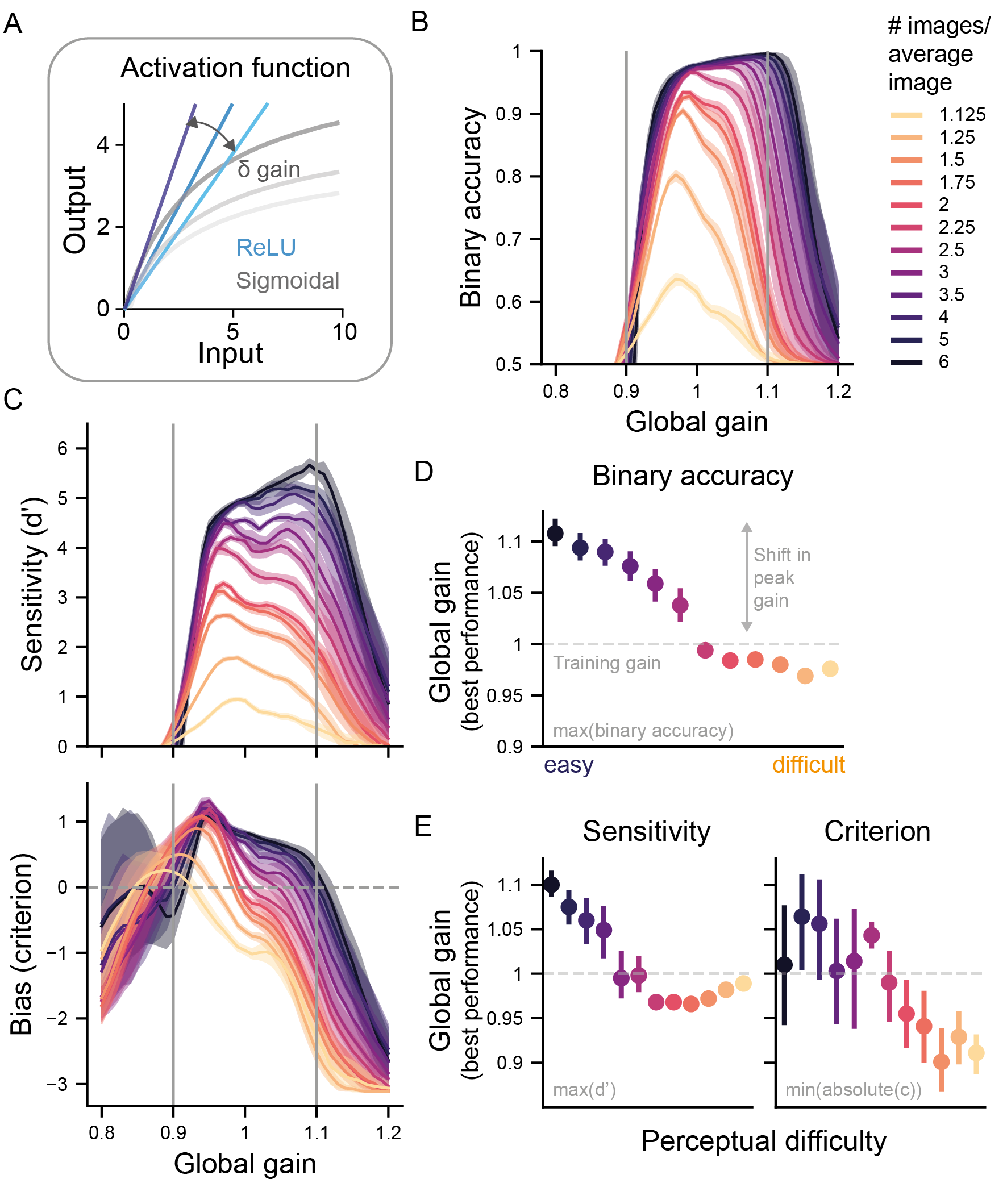

Supplement: S2 Fig — (A) Comparison of the activation functions used for the main experiments (sigmoidal) and an alternative (rectified linear unit, ReLU) evaluated in this figure. While activation regimes are comparable for small values, they diverge for large values. (B) Binary accuracy for all levels of perceptual difficulty as a function of global gain state. The shaded areas represent the 95% confidence interval (CI) across ten network instances in B and C. As in Fig 3, this pattern of results also reproduced the Yerkes-Dodson effect in the DCNN’s performance. (C) Signal detection properties as a function of perceptual difficulty and global gain. (D) Global gain level linked to peak performance per difficulty condition in B. In line with the Yerkes-Dodson effect, peak performance was again associated with reductions in global gain level with increasing task difficulty (as in Fig 3). The error bars correspond to 95% CI across ten model instances. If multiple gain states were linked to the best performance, the median was used to summarize them. The dashed line corresponds to the neutral gain state from training, during which no global gain changes are applied. (E) As in (C) but now global gain states linked to the performance for sensitivity and bias. (TIF) [file pcbi.1009976.s002.tif]

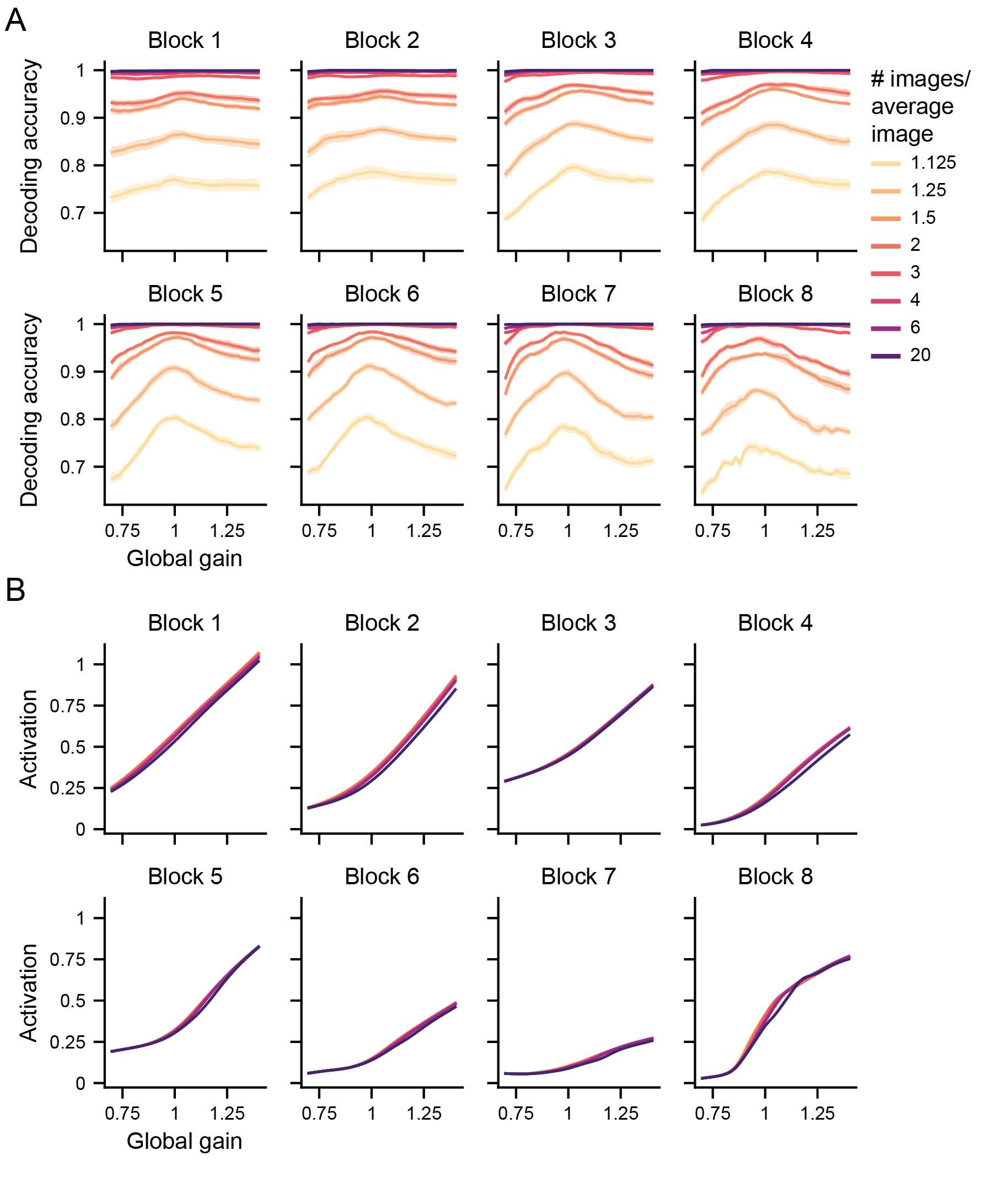

Supplement: S3 Fig — All figure conventions are the same as in Fig 5A. (TIF) [file pcbi.1009976.s003.tif]

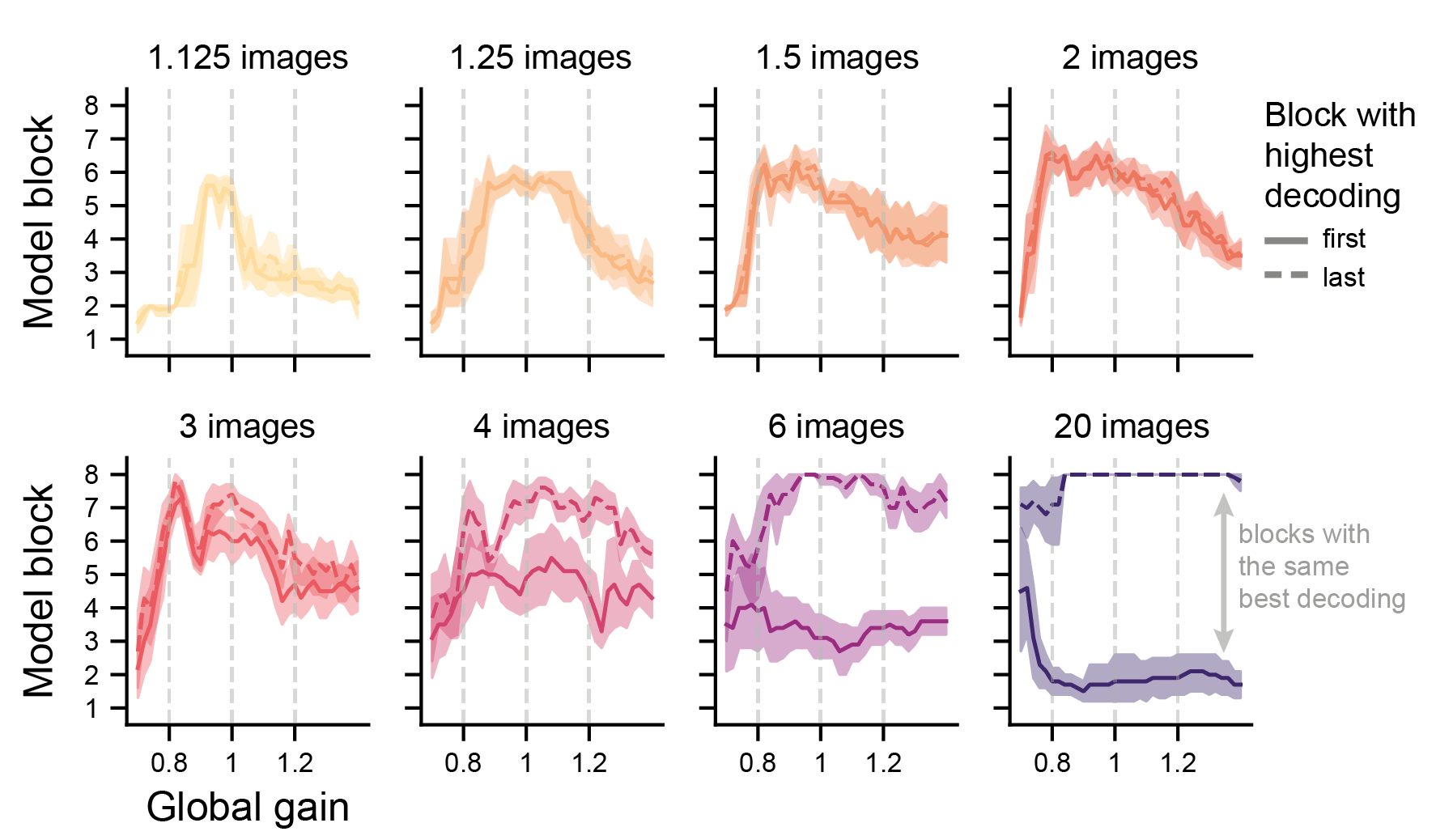

Supplement: S4 Fig — The vertical dashed lines indicate the data shown in Fig 4E and 4B. The shaded areas show the 95% confidence interval across ten network instances. (TIF) [file pcbi.1009976.s004.tif]

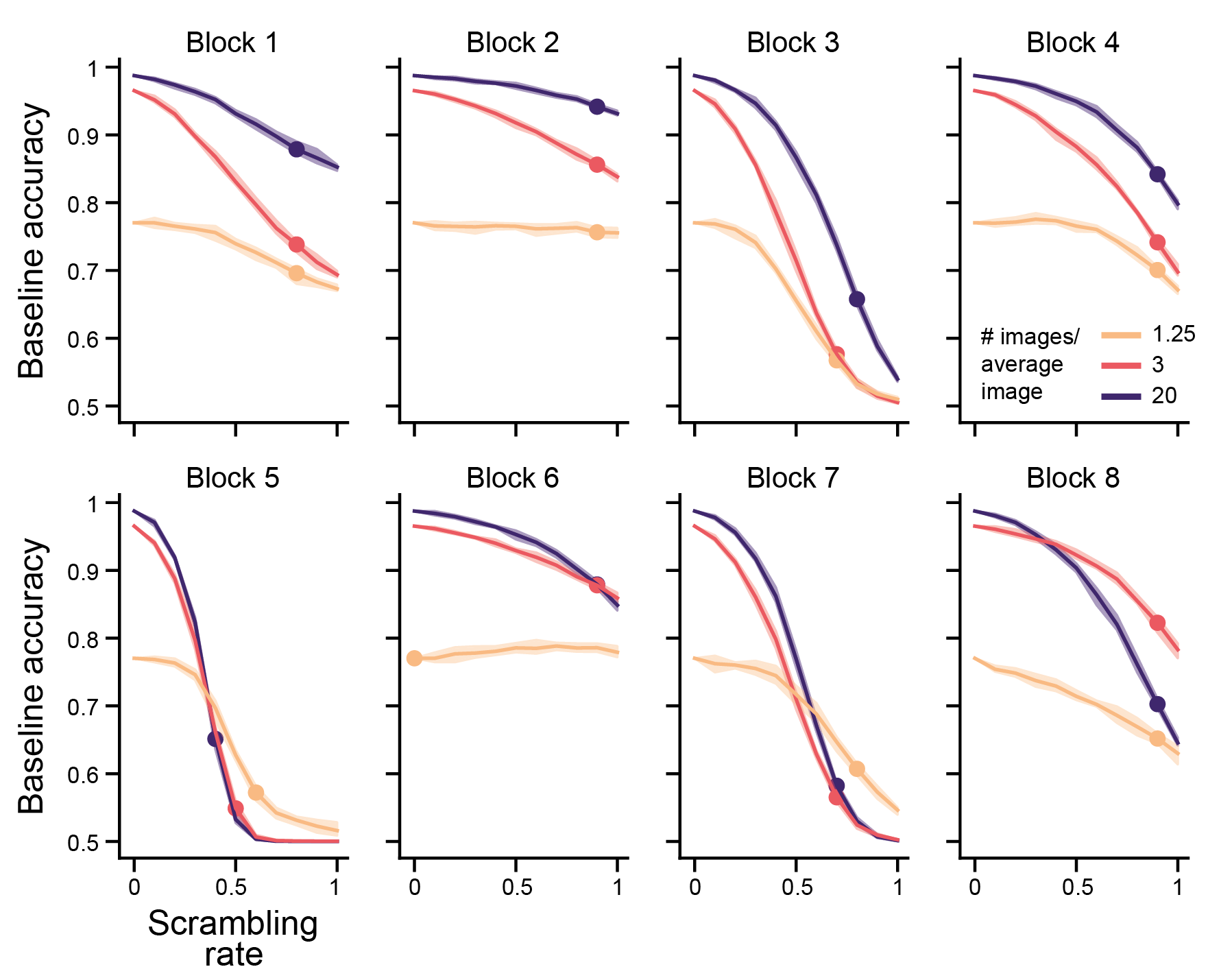

Supplement: S5 Fig — The dots represent the scrambling rate at which 20% of the baseline performance is maintained. (TIF) [file pcbi.1009976.s005.tif]

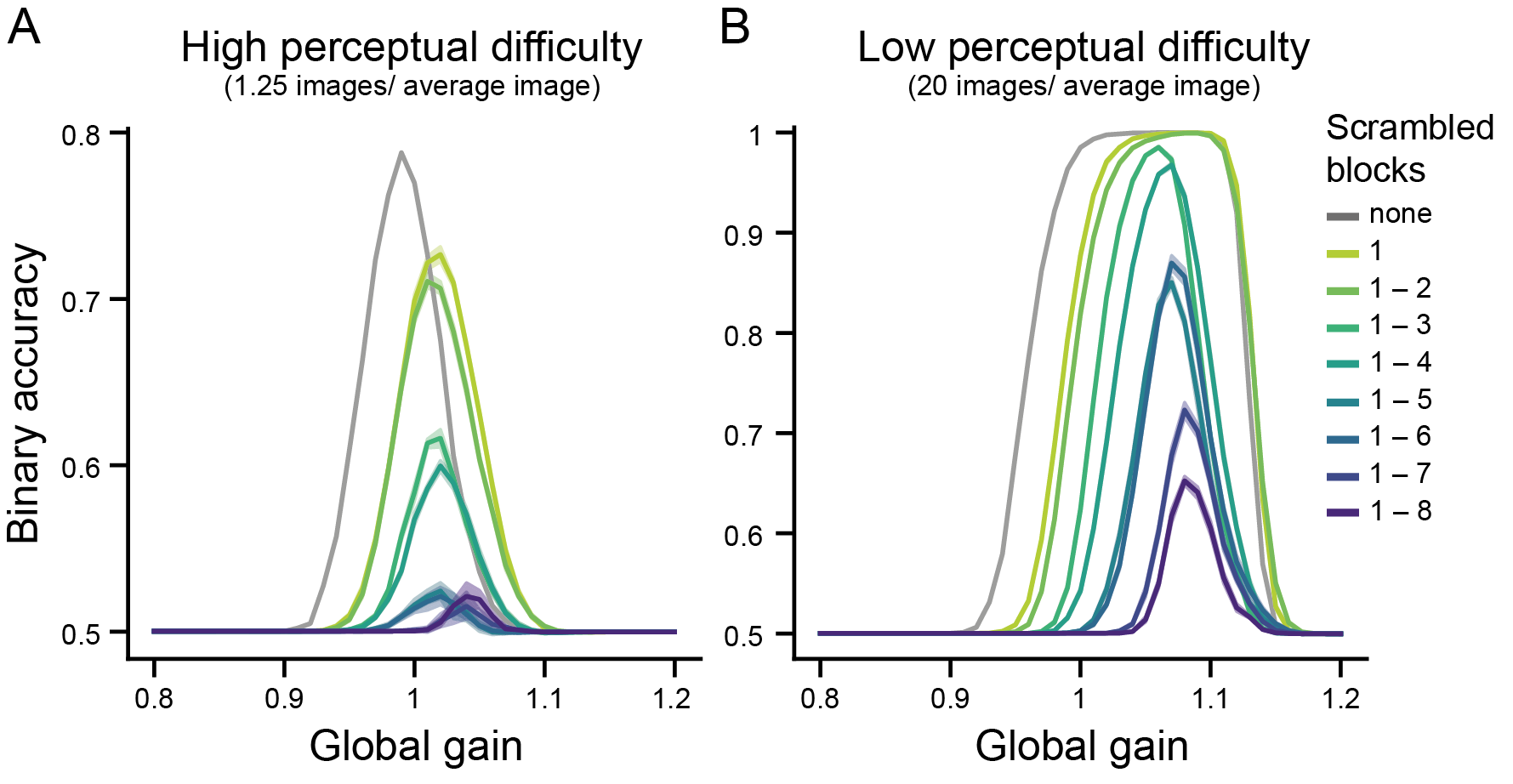

Supplement: S6 Fig — A shows a perceptually difficult task and B a perceptually easy task. All figure conventions are the same as in Fig 6. (TIF) [file pcbi.1009976.s006.tif]
