# Supplementary material for: Arousal state affects perceptual decision-making by modulating hierarchical sensory processing in a large-scale visual system model
Source: PLoS Comput Biol. 2022 Apr 4;18(4):e1009976. doi: 10.1371/journal.pcbi.1009976 (PMC9009767; doi:10.1371/journal.pcbi.1009976)
Supplement: Attachment — Submitted filename: ResponseToReviewers.pdf [file pcbi.1009976.s007.pdf]

Amsterdam, January 6, 2022

Dear Dr. Isik, dear Dr. Serre,

We hereby submit our revised manuscript "Arousal state affects perceptual decision-making by modulating hierarchical sensory processing in a large-scale visual system model" for consideration for publication in PLOS Computational Biology. Below is a detailed response to the comments by the reviewers, with the relevant parts of the original comments in italics, and our responses below that.

It was a pleasure to work through the reviews and the valuable comments. In particular, we added two key control analyses to the manuscript following the recommendation by the reviewers. These analyses further strengthen our original conclusions. Thanks to the reviewers' comments, our revised manuscript now also more clearly presents our results, including the associated figures. While looking into the reviewers questions, we have encountered additional relevant literature and updated our references accordingly. We have shared the updated versions of our code, and hope that this may be useful to the reviewers in evaluating our new analyses and their results. All in all, we believe our paper has improved substantially thanks to the reviews, and we hope that it is now suitable for publication in PLOS Computational Biology.

We look forward to your feedback and input on our revised manuscript.

Sincerely,
On behalf of all authors,
Lynn Sörensen

Responses to reviewer 1

We'd like to express our appreciation for the thoughtful and thorough appraisal of our manuscript by the Reviewer, which led us to conduct new analyses that we believe have substantially strengthened our paper and its original conclusions. We hope that we have fully addressed the reviewer's comments in the revised manuscript and in our responses below.

R1.1 *I'm concerned the title overstates the results, as it doesn't read as being model-specific. A small change such as "Arousal state can affect perceptual decision-making by modulating deep hierarchical sensory processing" would help.*

We thank the reviewer for raising this point and changed the title to be model-specific:
"Arousal state affects perceptual decision-making by modulating hierarchical sensory processing in a large-scale visual system model"

R1.2 *"Furthermore, stimulus-unrelated neural activity in early visual cortices is closely related to arousal [29]. This raises the intriguing possibility that arousal may affect task performance by modulating the processing of sensory features that perceptual decisions are grounded in." How does it follow that "processing of sensory features that perceptual decisions are grounded in" is what is modulated if it is *stimulus-unrelated* neural activity that is impacted by arousal?*

We agree with the reviewer that that formulation was confusing. We have reformulated these sentences in the revised manuscript (Introduction, p. 4).

R1.3 *The authors use two different style of tasks (detecting averages and cluttered classification) to claim that the Yerkes-Dodson difficulty effect is based on perceptual difficulty and not response difficulty. The use of only one type of task to represent each style of difficulty is insufficient to support this claim. If the authors want to make this claim they will need to test out other examples of perceptual difficulty and response difficulty. For example, they could use different levels of clutter with the same readout options to determine if clutter level (a form of perceptual difficulty) shows the Yerkes-Dodson effect. As another test of response difficulty, they could also use their averaged images to create a task where the network needs to determine how many images are in the average, with increasingly narrow ranges as options (for example, an easy*

binary test for greater or less than 5 images, and more difficult tasks to classify 20-40, 0-20, etc). Without testing more tasks, the authors need to significantly scale back their claims about perceptual versus response difficulty. Their results right now merely hint at the possibility that the Yerkes-Dodson effect is based only on perceptual difficulty.

We agree with the reviewer that we should be careful in claiming that the Yerkes-Dodson difficulty effect is based on perceptual difficulty not response difficulty, as that warrants confirmation with a larger battery of tasks. In our original manuscript, we already acknowledged that our model does not serve as a model for the brain as a whole, since DCNNs largely capture processing in the visual ventral stream, which could explain the selective effects of perceptual difficulty. In our revised manuscript, we now emphasize in several places that our findings should be extended to other tasks and difficulty manipulations in future studies:

"First, we observed that this finding may be specific to perceptual difficulty in our model, although extending this observation to more tasks in future work is necessary to ascertain this."

Results – Yerkes-Dodson effect is absent for another manipulation of task difficulty, p. 12

Furthermore, we have also adapted the discussion to also emphasize this better:

"We here found that the Yerkes-Dodson effect in our model could only be observed when manipulating perceptual difficulty, whereas our model was insensitive to another manipulation of task difficulty (i.e., number of answer options). This finding may seem at odds with perspectives linking the Yerkes-Dodson effect to manipulations of task difficulty more generally (e.g. [12–14,40,41]). Yet, there is long-standing debate about what the terms (emotional) arousal and task difficulty specifically denote, and both have been repeatedly criticized for their vagueness [12,13,41]. Indeed, recent studies on this topic relied mostly on difficulty manipulations in the perceptual domain [3,5,7,10,15]. Our results add to this body of work by suggesting that the interaction between arousal state and task difficulty may be selective to the perceptual domain. However, to firmly conclude this, these observations should be extended to other tasks and difficulty manipulations in future studies."

Discussion – DCNNs as a model for visual processing and perceptual difficulty, p. 22-23

R1.4 *It is interesting to see that at all task levels, an inverted-U is found. Could the claim that the model is reaching arousal states not seen experimentally be verified by comparing the amount of neuromodulation observed experimentally to these gain values?*

To our knowledge, arousal states are typically inferred on an intra-individual level based on correlated variables such as tonic pupil dilation (i.e., McGinley, David, and McCormick 2015; Neske et al. 2019; Podvalny et al. 2019; Reimer et al. 2016) or locomotion (Shimaoka, Harris, and Carandini 2018; Stringer et al. 2019). With this approach, participants are exposed to a task over longer periods of time and post-hoc, trials with varying degrees of, for instance, pupil dilation are binned into trials of low and high arousal, respectively. Thus, the range of pupil dilation observed experimentally determines the extremes of the studied arousal effects. In the paper we argue that "A biological system bound to homeostasis likely never visits such extreme arousal states", as we applied to our model. Following from this, very high arousal states are likely only observed very rarely. This perspective is a common argument for explaining variation in findings between empirical studies (Podvalny et al. 2019; Faller et al. 2019; Waschke, Tune, and Obleser 2019), because it remains challenging to obtain an unbiased measure of neuromodulation. So, in direct response to the reviewer's question, we do not think that the amount of global gain, as we have modelled it here, can be directly linked to the current experimental correlates of neuromodulation.

In our view, the most promising avenue to gain a better understanding of the full range of neuromodulation would be to manipulate an organism's arousal state via, for instance, pharmacological intervention (i.e., Gelbard-Sagiv et al., 2018; Pfeffer et al., 2021)) or more direct locus coeruleus stimulation. Yet, these studies too are currently limited in the number of arousal states they can experimentally examine (in a given study). Moreover, judging the feasibility of these approaches is beyond our expertise and it could very well be that there are other mechanisms at play, which may still prevent the animal from visiting extremely high or low

arousal states. Importantly, we hope that these questions will be addressed experimentally in the future and that our study may provide a motivation for that.

R1.5 *The authors claim "Since both datasets were curated to be challenging visual search tasks, they required the analysis of complex visual features. Based on this, one would expect an inverted U-shaped gain profile". But their own results suggest there is always an inverted-U, even for easy tasks, if gain is extended long enough. So this finding alone does not strongly support the idea that this is a perceptually challenging task.*

We completely agree with the reviewer that our formulation was unclear. What we meant here is that given that this is a perceptually challenging task, we expected the performance-gain profile to be centered on medium global gain states, in line with our findings in Fig. 3. If one adopted a limited global gain range (as done if Fig. 3B for illustrative purposes), then all conditions would be expected to display an inverted-U shaped performance-gain profile.

We have clarified this in the manuscript:

"These results also corroborate our findings on perceptual difficulty. Since both datasets were curated to be challenging visual search tasks, they required the analysis of complex visual features. Based on this, one would expect the best performance to be observed for medium global gain states (as was observed in Fig 3 for perceptually difficult tasks). This is indeed what we observed across all six conditions (both datasets, all answer options, Fig 4D)."

Results – Yerkes-Dodson effect is absent for other factors of task difficulty, p. 12

R1.6 *What are repetitions/error bars in Figure 5? I also am not clear on what 5C is.*

The error bars in Fig. 5 refer to the ten model instances (same as those analyzed in Fig. 3). Fig. 5C shows the average global gain value associated to the best decoding accuracy collapsed across perceptual difficulties (in contrast to Fig. 5D, which shows these values separately for different perceptual difficulties). To better highlight this, we have added a more descriptive title to Fig. 5C ("Average across perceptual difficulties") as well as revised the legend of Fig. 5:

(C) Model blocks feature most decodable task-relevant information at different global gain states across the hierarchy. The decoding peak refers to the median global gain values linked to the best decoding accuracy across model instances. To obtain an estimate per ResNet block, we averaged these values across perceptual difficulties (shown in D)."

Fig. 5, p. 13

R1.7 *I am curious about which layers contain the most task information relative to each other. It seems as though the middle blocks may be most useful for this average image task, and that could be important for understanding the results and why some layers may be more important than others. It may good to directly determine the layer at which decoding accuracy is highest for each combo of difficulty and gain, in order to explicitly show these trends. At the very least, all panels in 5A (and S2A) should have the same y-axis.*

We thank the reviewer for these excellent suggestions. We have accordingly updated the scaling of the y-axis in the Fig. 5A and S2. As suggested by the reviewer, we have also implemented an analysis that dissects which model block is most informative given a level of perceptual difficulty and global gain state (see Fig. 5E and S4). We believe that this analysis clarifies a central link that we only treated implicitly before, namely, that different blocks vary in their relative importance for solving tasks of different difficulties and that this relative importance, in turn, interacts with global gain. Thereby, this analysis complements our understanding of how global gain states interact with task difficulty more directly: Challenging tasks rely on more specific information located in later network blocks (in contrast to easier tasks), which is easily degraded when changing the global gain. In contrast, the relevant information for easy tasks, can be found across many network blocks and is rather robust to changes in global gain.

We have added an elaborate description of this analysis to the results and adjusted our conclusions:

"These results are corroborated by an analysis, which assessed the relative importance of these blocks and showed that in a medium, that is, neutral global gain state (middle panel, Fig 5E), most tasks were best solved relying on information of later network blocks (blocks 5-6). While this also applied to easier tasks, such tasks were also equally well solved based on information of either much earlier (i.e., block 2, circles) or later model blocks (i.e., block 8, diamonds). Thus, information used for solving easy tasks was spread out along the model hierarchy, whereas for challenging tasks, such information was limited to a small subset of blocks. Interestingly, when global gain changes were applied to the network, this pattern mainly changed for the difficult tasks (left and right panel, Fig 5E): for both low and high global gain states, the most informative block was no longer located late in the hierarchy (block 5-6) but instead markedly earlier (block 2 & 3). This suggests that later model blocks were no longer as informative for solving difficult tasks when put in these altered global gain states. In contrast, the easier the task, the less global gain changes appeared to modulate the location of the most informative block along the model's hierarchy.

Taken together, we found that different stages of hierarchical processing were associated with different optimal global gain states for encoding task-relevant information and that the relevance of these stages was strongly modulated by perceptual difficulty. While global gain states facilitated sensory encoding in early network blocks at high gain states, late network blocks contained most task-relevant sensory information at medium gain states, that performance on perceptually difficult tasks more strongly depended on than performance on easy tasks."

Results – Global gain differentially affects task information across the model hierarchy, p. 14-15

R1.8 *On the whole, I do not understand what the authors believe their scrambling analysis achieves or how to interpret these results. In the Methods they say "we either gradually disrupted later model blocks first and successively added earlier model blocks or the other way around, thereby either leaving mostly earlier or later model blocks intact" Scrambling early blocks does not leave later blocks intact (as their activity relies on the activity patterns in the earlier blocks). Also, keeping the early blocks constant and scrambling later blocks does not allow us to know the impact of early blocks, as that impact is mediated only through the (now scrambled) later blocks (even in the presence of skip connections, the network is still relying on the normal arrangement of the residual inputs to create activity patterns at the next layer; it can't dynamically choose to rely on the skip connections because the residuals are scrambled). I simply don't know if the attempt to ascribe different task performance to different layers is sensible in a hierarchical network. The activity at any given layer only matters for classification to the extent that it can modulate the activity that comes between it and the classifier. In this way the features at different layers aren't separable. I think the decoder analysis is interesting but for similar reasons it also doesn't actually tell us which layers are most important for performance increase at specific gain levels (i.e. what you can readout at a given layer under a given gain condition may not matter if that information has been extensively transformed by the time it actually gets to the classifier layer). It seems like a better framing would be to discuss what effect the early layers have on later layers, such that early layer features can impact performance.*

We understand the reviewer's concerns and agree that, in principle, one cannot change part of the processing in a hierarchical network without changing all subsequent levels of processing. We have hence rephrased our wording on this in the revised manuscript in corresponding places to point out that, as in any complex biological system, manipulating one part of the system also affects the functioning of other parts of the system (p. 17 of the Results section and p. 30-31 of the Methods section). Yet, system perturbation is a common approach to study causal factors in the attempt to understand complex systems such as biological brains (i.e. Wischnewski and Peelen 2021) and can be particularly useful if strong hypotheses can be tested. We have here accepted some of the fundamental flaws and limitations that come with this approach (as outlined by the reviewer), so as to directly test our hypotheses that the changes in the network's representations directly affect the network's performance. We deemed this crucial so as to exclude the possibility that the decoded information (see Fig. 5) that we analyzed was not relevant to the network's performance. After all, the network learns a set of non-linear transformations on these representations and is therefore not limited to linear decoding.

Yet, we agree with the reviewer that network layers cannot be understood in isolation. Indeed, this concern was also our motivation for implementing a scrambling analysis in the first place, rather than, for instance, using a drop-out approach for interfering with the network. With dropout, randomly selected weights or units are dropped at a specified rate, leading to quite dramatic changes in the network's activation distributions. In contrast, spatial scrambling along the spatial dimensions, as we have used, maintains the distributional properties of the activation along the feature dimension. So, while our aim was to impair a given block's outputs, we did not want to cause large distributional shifts in the network's activation. Such distributional shift may make the network fail for trivial reasons, such as mismatch with the batch normalization statistics. We were not interested in these failures, but rather those specific to changes in the information in a given ResNet block. Importantly, we believe that residual neural networks are particularly suited for this with their skip connections, allowing the unscrambled information to propagate through the network to still reach later model blocks. We agree with the reviewer that also these later blocks partly rely on the inputs of earlier blocks; however, the skip connections also make it possible for some of these later features to be driven more directly by information from the network's encoding stages. That is to say that ResNets, in part, abstract away from the strict hierarchy of features and enable information to flow more flexibly. In our view, spatial scrambling in combination with the residual connection enabled us to establish a more nuanced comparison of the relative importance of different network blocks and how they vary across gain states to performance. This nuanced quality of interference of our spatial scrambling analysis can be, for instance, seen in Fig. 6B as well as in Fig. S6, where targeting an additional network block leads to a continuous decrease in performance. Adjusting the rate of spatial scrambling to a specific performance drop (Fig. S5) was another way to control this process. Importantly, this allowed us to compare the same networks across a wide range of spatial scrambling states, global gain states and perceptual difficulties, systematically teasing these influences apart, a key advantage of a causal manipulation.

To summarize, the reviewer raised a valid point related to the general difficulty of studying parts of complex biological systems in isolation, which we acknowledge in the revised manuscript. Nonetheless, in our view, our scrambling approach importantly added to our decoding results by allowing us to test specific hypotheses on the relative importance of different model blocks in driving the observed interaction between global gain state, task difficulty and hierarchical processing.

R1.9 *I also don't understand the attempt to relate the amount of gain needed under different scrambling to which layer the network is relying on under different gains in the normal networks. The authors say "Our results so far suggest that high global gain states optimize sensory processing in early blocks. Therefore, we expected that increasingly disrupting late network function (Late to early) and giving more weight to earlier blocks, would lead to a right-ward shift in the performance-gain profiles" You could also expect the opposite: if it is relying more on early layers maybe early layers wouldn't need to be as "loud" as when it's competing with influence from later layers. As far as I can tell there is also a different logic at play here: "Only once also the very last network features were affected, there was a notable increase towards higher gain states for peak performance on the difficult task. This pattern of results suggests that sensory processing in later network blocks is more critical for solving perceptually difficult tasks and that medium global gain states facilitate later processing and hence performance on difficult tasks. " This is saying that if the layer that is important for performance (here, higher layers) is scrambled the network will need higher gain, whereas the first statement seems to say that if the layers *not* needed for performance (also higher layers) are scrambled, the network will need higher gain. The authors need to clarify this seeming contradiction.*

We thank the reviewer for this careful analysis of our argument. We are not completely sure that we fully understand the contradiction pointed out by the reviewer, and we hope that we have correctly interpreted and addressed this comment below.

While rereading these paragraphs that the reviewer refers to, we realized that the current focus on the contribution of single network blocks may conceal that under all circumstances all network blocks are contributing to the network's performance and thus performance-gain profiles. Based on the analyses presented in Fig. 5, we hypothesized that the interaction between task difficulty and global gain state apparent in performance was driven by the fact that features along the hierarchy were differentially important for different task difficulty and that these features, in turn, were best encoded under certain global gain states (Fig. 5C). The assumption behind this analysis was that all blocks jointly shape the performance-gain profile and the associated peak global gain state. If, however, we degrade feature information in a given block via

spatial scrambling, these features become less informative for performance, irrespective of the global gain state of the network, and as a result, will contribute less to the performance-gain profile. So rather than a competition as mentioned by the reviewer as an alternative interpretation, this analysis is always bound to be a sum of all effects. More specifically, what we meant with "*giving more weight to earlier blocks*" was that the *relative* importance of these features will grow in shaping the performance-gain profiles, given that, for instance, later blocks become less informative.

Based on the reviewer's comment, we do however see how the idea of a competition between feature blocks may arise based on the description of the second quoted result. Indeed, our wording leaves room for the interpretation that higher global gain states serve as a compensation for an increasing number of layers targeted by spatial scrambling. This is however not what we intended to express: rather, that for difficult tasks, higher network block features are more important and thus more strongly shape the model's performance-gain profile by linking the best model performance to medium global gain states. If, however, these features are scrambled, this influence diminishes and the sum of all other blocks (both scrambled and intact) becomes more prominent. So, from this perspective, the best performance for the difficult task was associated to medium global gain states, as long as these late network features were intact and informative for performance.

Initially, we expected that the later a network block in the model hierarchy, the more important this block's features would be for solving difficult tasks. The results shown in Fig. 6D for the difficult task are inconsistent with this initial expectation in that scrambling block 7 and 8 led to a shift to *higher* global gain states, instead of further keeping the global gain profile constant (as seen for scrambling block 1-6). Interestingly, this result may be clarified by the suggested decoding analysis (see ***R1.7***), by showing that the most task-relevant information for the difficult task could be found in block 5 to 6 for neutral global gain states, rather than in the last blocks. This means that as long as the model block 6 (i.e., the block most informative for this task difficulty level) was intact, the network performed best at lower global gain states. Beyond that network features were less relevant and thus did not relatively matter in the sum of all network blocks.

We hope that our explanation addresses the reviewer's comment. In line with the points raised, we have adapted the description of this analysis and its results:

"Selectively controlling the contribution of a model block to the overall performance is a challenging problem, since the functioning of a network block depends on the activation distributions of the preceding network blocks. To address this, we developed the spatial scrambling method (see Fig 6A). This approach selectively perturbs the information for a given block, but maintains its activation distributions. As a result, this model block's features contribute less effectively to performance and the remaining, unaffected blocks will consequently dominate the model's performance (see methods for an in-depth description of spatial scrambling). Importantly, spatial scrambling led to a relative reweighting of feature contributions to performance. That is, under all circumstances, all network blocks were still affecting performance, but features that were scrambled were less informative and thus exerted less influence on the performance-gain profile. We leveraged this principle to dissect the network's performance, and determine how the features along the model hierarchy differentially contributed to performance of easy and difficult tasks. Specifically, we either increasingly added spatial scrambling from the top to the bottom of the network, thereby making the network more and more reliant on feature information contained in the early blocks or we applied it from the bottom to the top of the network, so that late network features drove performance (see Fig 6A).

Our results so far suggest that high global gain states benefit sensory processing in early blocks. Therefore, we expected that increasingly disrupting late network function (Late to early), i.e., assigning more weight to earlier blocks, would lead to a right-ward shift in the performance-gain profiles. That is, we expected to find the best performance during higher global gain states in this case, since early features are encoded well during high global gain states. Fig 6B shows that this expectation was met for both perceptually difficult and easy tasks. In particular, we observed that greater reliance on processing in early network blocks led to peak performance at higher global gain states (see Fig 5C, Late to early). Notably, this resulted in very high global gain states compared to the baseline network (see Fig 6B and C). Across both task difficulty conditions, peak performance shifted to the highest global gain states if almost all late network features were scrambled. These findings suggest that higher global gain states may facilitate network performance by enhancing

feature content in early network blocks. Beyond this general effect, there was also a difference between task difficulty conditions: As perceptually easy tasks relied more on sensory processing in early network blocks than perceptually difficult tasks, scrambling late network blocks had less of an effect on performance on easy tasks, as shown in Fig 6B.

In the absence of spatial scrambling, we observed that in contrast to easy tasks, the network's performance on difficult tasks was best at medium global gain states (Fig 3). As we found optimal decoding of task-relevant information in later blocks at medium global gain states for difficult tasks (Fig 5), we expected that first scrambling early blocks would not substantially affect the relationship between gain and performance on difficult tasks until scrambling also included later blocks. Here we assumed that in particular these later network features determine the performance-gain profile, since these features are specifically linked to high performance at medium global gain states. To test this, we next scrambled the network blocks increasingly from early to late blocks (see Fig 6C, Early to late). Indeed, the global gain state associated with the best performance remained relatively stable and visibly lower compared to the early-to-late condition for both task difficulty levels (see Fig S6 for performance-gain profiles). Only once also the very last network features were affected (block 7 and 8), there was a notable increase towards higher gain states for peak performance on the difficult task. This finding indicates that in particular information contained in block 6 was important for this task, biasing the performance-gain profile towards lower global gain states. This conclusion is also in line with the decoding result suggesting block 5-6 to hold most task-relevant information for difficult tasks. Taken together, we find that sensory processing in later network blocks is more critical for solving perceptually difficult tasks and that medium global gain states facilitate later processing and hence performance on difficult tasks. In contrast, easy tasks rely more on sensory processing in early blocks, which operate better at higher global gain states."

Results – Task difficulty differentially engages model features across the model hierarchy, p. 17-19

R1.10 *The statement "Our results so far suggest that high global gain states optimize sensory processing in early blocks. Therefore, we expected that increasingly disrupting late network function (Late to early) and giving more weight to earlier blocks, would lead to a right-ward shift in the performance-gain profiles" also seems to suggest that you would expect to *not* see such a rightward shift for the difficult task (as Figure 5D shows an optimal gain around only 1 there). Yet the same rightward shift occurs.*

In line with our response to ***R1.9***, we did not want to suggest with our description that early network features do not matter for difficult tasks. Rather, we believe that the performance-gain profile is a sum of all effects of global gain along the network hierarchy. So, relatively, interfering with late network blocks will also lead to a shift in the performance-gain profile, since earlier network blocks would still feature more relevant task information at higher global gain states.

We have added a statement to the results to better highlight this:

"Importantly, spatial scrambling led to a relative reweighting of feature contributions to performance. That is, under all circumstances, all network blocks were still affecting performance, but features that were scrambled were less informative and thus exerted less influence on the performance-gain profile. We leveraged this principle to dissect the network's performance, and determine how the features along the model hierarchy differentially contributed to performance of easy and difficult tasks."

Results – Task difficulty differentially engages model features across the model hierarchy, p. 17

R1.11 *I think a more fruitful analysis to understand the role of activity at different layers for easy versus hard tasks may be to simply modulate the gain at different layers individually. It is conceivable, for example, that difficult tasks do rely more on the features at later layers, but the model only needs low to medium gain to activate those layers strongly because the later layers are inheriting impacts from gain changes at earlier layers. Changing gain at individual layers may isolate some of these effects. It may also reveal different behavioral patterns. For example, the authors note that criterion doesn't have a U-shape with gain. In Lindsay & Miller (eLife, 2018) (fig 5E) gain changes (as a model of spatial attention) create a U-shape in criterion when applied at early layers but not at later. Given the sigmoidal activation function used in this*

model, the propagating impact of gain changes at one layer on later activity and classification is even harder to predict without direct experimentation.

We thank the reviewer for this interesting suggestion. We are hesitant to investigate the results in the networks' performance with this approach for multiple reasons: First, we do not have specific hypotheses or expectations what would happen if we would apply global gain to the network blocks individually, making the interpretation of the findings of any causal manipulation problematic in our opinion. Second, it was our goal to understand how the performance pattern arose while applying global gain to all network blocks. In our view, applying gain to a single network block at a time, would create dramatically different initial conditions and it may be hard to see how to compare these scenarios. Third, our implementation of arousal as global gain has a strong empirical basis, whereas, as mentioned by the reviewer more local and targeted gain manipulations have been previously linked to other phenomena, such as selective attention (Lindsay and Miller 2018; Sörensen et al. 2020; Luo, Roads, and Love 2020).

R1.12 *I'd like to see the authors discuss in more detail (as well as give examples of) what tasks have been considered "easy" versus "difficult" in the experimental literature and how their tasks relate to these concepts as they are used experimentally.*

Following the reviewer's recommendation, we now elaborate on task difficulty and how our task relates to other experimental tasks in the discussion:

"The Yerkes-Dodson effect was initially observed with a black and white visual discrimination task and task difficulty was manipulated by varying ambient light during a learning task [11,61]. For the current study, we chose a manipulation of task difficulty that mimicked those of more recent studies [2,3,5,7,10], which varied the signal-to-noise ratio (SNR) of target stimuli, with tasks with a higher SNR considered easier. In those studies, the SNR is usually increased by varying the target's intensity, for instance, its contrast. While our approach of averaging a different number of images (see Fig 2) also varies the SNR, we did not vary the images' contrast, and this may be an important avenue for follow-up experiments. In a similar vein, many different stimuli have been used for studying the effects of arousal state on perceptual processing: Some studies also used naturalistic stimuli as targets [5,7,10], as we did here, whereas others used more artificial stimuli such as Gabor patches [5], moving dots [15], drifting square-wave gratings [2] or pure tones [3] as target stimuli. Our observation of an interaction between global gain state, perceptual difficulty, and the model's processing hierarchy predicts that tasks that rely on feature distinctions of varying complexity should also have a different relationship to arousal state. Importantly, the current modelling framework makes it possible to assess the interaction between stimuli, task difficulty and arousal state more systematically in future studies."

Discussion – DCNNs as a model for visual processing and perceptual difficulty, p. 23

R1.13 *I'd also like to know how using the same gain factor at all layers correspond to biologically data? i.e. does global arousal impact V1-IT to the same extent or are there differences in different areas?*

Available data in this area does not yet provide a direct answer to this question, since there are very few studies in non-human primates, which specifically examined this (see (McCormick, Nestvogel, and He 2020) for a recent summary of this). A research area closely linked to that of arousal is cortical state: In this area, there have been some recent studies examining cortical state changes in V1 and V4 in non-human primates (Beaman et al., 2017; Cowley et al., 2020; Engel et al., 2016; Gutnisky et al., 2017) and some have reported a link to arousal (Cowley et al., 2020; Engel et al., 2016).

While there is few data in non-human primates and in particular on hierarchical interactions, decades of research in rodents along with many neuroanatomical studies across species support the idea of similar effects in along the visual ventral stream (incl. IT). Across species, it is well established that LC and its noradrenergic nuclei project to virtually all brain regions, including sensory cortices (Sara 2009; Berridge and Waterhouse 2003). This makes it likely that these modulation are uniform and unspecific (i.e. McGinley et al. 2015; Poulet and Crochet 2018; Aston-Jones and Cohen 2005; McCormick, Nestvogel, and He 2020; Sara 2009). Furthermore, these projections have been shown to lead to gain changes on a cellular level (Sara

2009; Polack, Friedman, and Golshani 2013), to covary with pupil dilation (Reimer et al. 2016) as well as to lead to an increase in signal-to-noise ratio in rodents (Reimer et al. 2014; Polack, Friedman, and Golshani 2013; Vinck et al. 2015). Finally, while most current evidence on the effect of neural processing stems from mice (Stringer et al. 2019; McGinley, David, and McCormick 2015; Reimer et al. 2016, 2014; Vinck et al. 2015; Neske et al. 2019), these effects are not believed to be specific to mice (Sara 2009; Aston-Jones and Cohen 2005; Berridge and Waterhouse 2003).

We have added a sentence to the discussion to highlight this current gap in knowledge:

"However, while there is a growing body of evidence for arousal effects on sensory processing in mice [2,3,25,26,29,47], non-human primates [58,59] and humans [5,7,10], to our knowledge, it is still unclear how arousal state impacts higher visual cortices essential for object recognition and how those effects may be dependent on the visual task at hand. We hope that our model can serve as steppingstone for experimentally investigating these open questions."

Discussion – DCNNs as a model for visual processing and perceptual difficulty, p. 21

Responses to reviewer 2

We want to thank the reviewer for their encouraging words on our paper and their constructive feedback. Working through the reviewer's questions and suggestions has substantially strengthened the manuscript in our view, accounting for a large potential confound. The revised manuscript incorporates most recommendations put forward by the reviewer and we hope to have addressed all concerns in a satisfactory fashion.

R2.1 *The task difficulty effects are, in my mind, quite interesting. That said, I'm not sure that the authors have fully explained how they come about – and within this simplified framework, it should certainly be possible. I would venture to guess that the distributions of input magnitude experienced for units at the different levels of difficulty drive the effects – simply put, trials where inputs are a small range of values, or when inputs are all very large, pushing outputs onto the flat part of the activation function – should yield poor performance. So if a given stimulus set (ie. difficult) tends to lead to very high inputs at any cortical layer, then decreasing gain could reduce inputs, and push the input distribution onto the steepest part of the activation function. Is this what is going on here? It seems very testable. For starters the authors could look at overall activity levels for each layer separately for the different difficulty conditions… do they differ? How are they affected by the gain manipulation? Are they just being pushed toward the steep part of the activation function by the best gain? For a given layer, what is the distribution of input received for a given level of difficulty – and where does that fall relative to the activation function? If the activation function from the previous layer were turned down, would that decrease the inputs to this layer and push things onto a steeper part of the activation function? In my mind, the advantage of this model system is that these questions are answerable – and thus it makes sense to see how exactly the effects emerge, rather than speculating a mechanism based on layer specific decoding results.*

We thank the reviewer for these interesting points and questions. A straightforward way to address them is to replace our current sigmoidal-like activation function with a more standard rectifying linear unit activation function (ReLU, Nair and Hinton 2010). While we initially chose the sigmoidal-like activation function for its increased biological plausibility, repeating our experiments with a ReLU takes away the issue around the difference in steepness linked to different numerical ranges raised by the reviewer. With a ReLU, activation cannot be pushed into steeper parts of the activation function, since the slope is identical for all positive values in a ReLU. Our results can be inspected in Fig. S3 in the revised manuscript. In brief, we fully replicated our earlier pattern of results: While easy tasks succeed at very high gain states, more challenging tasks perform best at medium global gain states. This implies that our earlier results are not due to activation levels being compressed due to changes in gain, but rather, that features across the model hierarchy are differentially affected by these changes in gain, a feature that is shared between models with a ReLU and a sigmoidal-like activation function. We hope that this additional analysis alleviates the reviewer's concerns regarding the activation levels and distributions that markedly change as a function of gain.

We now also mention this additional analysis in the results section:

"As mentioned above, we here adopted a biologically-inspired activation function in our global gain model. However, most DCNNs feature a rectified linear unit (ReLU, [39]). Since global gain directly acts on the activation functions, the shape of this function might deeply matter for the observed effects on the network's performance. For instance, global gain could simply compress the activation at high levels of activation due to the shape of the activation function (see Fig 3C for an illustration), which cannot happen with a ReLU, since it is fully linear in the positive range (see Fig S2A for a comparison). To determine the specificity of our findings, we repeated the same experiments as presented in Fig 3, but with a DCNN featuring ReLU units. Crucially, we fully replicated our findings (see Fig S2). This indicates that the Yerkes-Dodson effect observed here is not a by-product of applying global gain to a specific activation function, but rather reflects a global gain mechanism acting on a sensory processing hierarchy of learned perceptual features."

Results – Yerkes-Dodson effect is robust to the shape of the activation function, p. 10

R2.2 *If all of these effects are driven by changes in the overall activity levels in different layers, it also suggests that these effects should be extremely sensitive to normalization. In the most extreme case, if inputs were normalized to be in the same range for all difficulty levels, then these effects should go away, right? I'm less clear on what happens if outputs are normalized, rather than inputs (ie. divisive normalization), but in the case where these effects are driven by changes in overall activity/input levels, this definitely seems like it should be considered. A second issue that arises if overall activity changes substantially with different level of gain is that this is, to the best of my knowledge, not what happens in the brain. Instead some neurons increase their activation and others decrease their firing (Devilbiss & Waterhouse 2004, Devilbiss & Waterhouse 2011). Both of these issue could potentially be addressed through a different activity function in which gain is not monotonically linked to overall activity/output.*

We thank the reviewer for these pointers. One of the expectations we had prior to implementing this global gain mechanism in its current form was an increase in activation levels since this had also been observed in neural data for task-evoked activity in early visual cortex (i.e., Fig. 3 and 4 in Neske et al., 2019, Vincken et al., 2015). Furthermore, assuming a change in the slope of the activation function has been a common assumption in other modelling work (i.e., (Servan-Schreiber, Printz, and Cohen 1990; Usher et al. 1999)). As a result, observing that our global gain mechanism brought about differences in activation levels, was our predicted outcome and we did not see it as a confound that should be normalized away.

We read the suggested papers by Devilbiss & Waterhouse (2004, 2011) with great interest and agree that our current depiction of the effects of arousal on neurons may most likely be an oversimplification, as is most modelling work attempting to link neural mechanisms to task performance. We now point to this in the discussion (p. 20, Discussion – DCNNs as promising avenue for modelling the effects of cortical arousal). With regard to the more specific observations in Devilbiss & Waterhouse (2004) for somatosensory processing and how they connect to our study, recent work suggests that arousal has qualitatively different effects in somatosensory and primary auditory or visual cortices (Shimaoka, Harris, and Carandini 2018). Leveraging widefield voltage imaging, Shimaoka et al., 2018, concurrently monitored primary visual, auditory and somatosensory areas across changes in arousal state (in this study operationalized as locomotion). They replicate the earlier findings by Devilbiss and Waterhouse (2004) by observing a hyperpolarization in primary somatosensory cortices after movement onset. For primary visual and auditory cortex, however, they report a depolarization suggesting facilitation and increased responsivity, in line with an increase in gain as we have modelled here. This is also in line with a range of other studies in early visual cortices showing an increase in evoked responses with increases in pupil dilation (Vinck et al. 2015; Neske et al. 2019), a close proxy for arousal state mediated via noradrenaline (Reimer et al. 2016). These latter studies served as the main empirical basis for our modeling choices.

R2.3 *In general, the results here seem like they would be highly conditioned on the specific details of the activation function. This should be explored and the activation functions used for simulations should be better explained. Based on the figures, the slope of the activation is steepest for small levels of input – but this seems like it would be a poor match to actual cortical pyramidal neurons, which typically require many*

simultaneous synaptic events to hit spiking threshold. On the other hand, if the key results are robust to the specific sigmoidal function, I would worry less about these details.

We fully agree with the reviewer that it is crucial to explore other activation function as we did in the updated version of the manuscripts, now also replicating our findings using a ReLU activation function (see Fig. S2). We have also added a more elaborate description of the sigmoidal-like activation function to the updated manuscript:

"The second part of the equation describes the sigmoidal-like properties of the activation function where the constants h, c_0, c_1, c_2, c_3 and c_4 were derived from a spiking neuron model, capturing the mapping between the incoming current and the average postsynaptic potential over infinite time steps [32]. In brief, we changed the global gain of our model with a single parameter \delta, which scaled the output of the activation functions, thereby increasing or reducing response gain everywhere in the model.Note that an activation function according to this formulation is relatively comparable to a ReLU for small values but exhibits large differences for large input values due to its sigmoidal-like saturation (see Fig. S2 for a visual comparison)."

Methods – Global gain mechanism, p. 26

R2.4 *In my mind, the fact that a CNN trained using a single gain will show performance that follows an inverse U function of gain, centered on the training gain, is not particularly surprising. This is what is supposed to happen – the model learns weights that are appropriate for trained activation function – if the activation function is changed, performance will suffer, to a degree that depends on the size of the change. In a sense, this is an interesting example of state dependent learning – which actually could be a high-level theory for the Yerkes Dodson inverted U, but probably doesn't require a sophisticated model. Thus, I see figure 4 as suggesting a limitation of this modeling approach, rather than presenting evidence for the explanatory potential of gain in a feedforward network.*

We agree with the reviewer that it is not surprising that performance is decreased when a model needs to generalize beyond its training. Surprisingly, however, we observe that this expectation was not met for perceptually easy tasks (Fig. 3). Instead, we observed that high gain states benefit performance on these tasks. This means that the model for these situations generalized and even improved compared to its training. We therefore believe that the key contribution of this work is that these effects arise in a hierarchical sensory system. That is, a system that has been optimized on a challenging visual task, such as object recognition, and therefore needs to develop a representational hierarchy. Our contribution is that we highlight that such a sensory hierarchy may differentially interact with dramatic changes in gain and hence shape performance.

We now highlight this in the results section:

"One could argue that the observed inverted U relationship between gain and performance, centered on the neutral gain value of 1, simply reflects state-dependent learning, as the models were trained and fine-tuned in a neutral gain state of 1. However, arguing against this alternative account, the observed pattern of results in Fig 3A shows that the models not only generalized beyond their trained global gain state by achieving similar levels of performance, but critically, that model performance at easier tasks was even better at higher global gain states. This not only displays the models' robustness towards global gain changes, but also suggests a computational advantage of high global gain states for easier tasks."

Results – A DCNN with global gain replicates the Yerkes-Dodson effect in performance), p. 8-9

R2.S1 *Figure 6 could use some additional explanation. The x-axis should be clearly explained in the legend. I also was not clear why the authors chose this particular manipulation – rather than one that would be more selective for the gain manipulation (ie. changing gain of one layer at time while leaving others fixed).*

We thank the reviewer for catching this, we indeed did not provide an explanation of the x-axis in Fig. 6C and D. In the updated version of the manuscripts, we have added this information:

"(C) (…) The x-axis shows the progression from few to many blocks being scrambled, and in this case, starting from late and progressing to earlier blocks (as is also indicated by the color legend in B). (D) (…) The x-axis is as in (C) but now progressing from early to later network blocks."

Fig. 6, p. 16-17

Our motivation for applying gain in a global fashion was that we wanted to understand the origin of our performance findings (Fig. 3), where we also applied global gain to all network blocks. In our view, applying gain to a single network block would potentially create an incomparable situation. In contrast, the spatial scrambling allowed us to also interfere with the network's processing in a targeted and gradual fashion, while keeping the global gain as well as the perceptual difficulty manipulations intact. We have adapted the manuscript to better highlight our motivation for choosing this method:

"In the last analysis, we have shown that also decodable information is associated with different optimal global gain states across perceptual difficulties. Crucially, this link varied along the model hierarchy. This observation begs the question whether this linearly decodable information is actually used during network performance. After all, during training, the network learns a set of non-linear transformations on this information and is therefore not limited to linear decoding for performing on this task. To address this question, we devised a causal manipulation that allowed us to selectively reduce the contribution of each model block to the network's performance. In particular, we took advantage of the architecture of our model that has two main branches, one for maintaining and one for processing information (see Fig 6A). By interfering with the information in the processing branch, we were able to inspect the performance-gain profiles with normal and disrupted block function. Like this, we could assess whether feature information in early blocks during higher global gain states is associated with improved performance on easy tasks, as well as whether feature information in later network blocks during medium global gain states benefits model performance on difficult tasks. In brief, we find that indeed early network blocks are the main driver behind optimal performance during high global gain states for easy tasks. Furthermore, this analysis makes it evident that the difficult tasks particularly rely on late network features for performance, which biases them towards medium global gain states in contrast to easy tasks.

Selectively controlling the contribution of a model block to the overall performance is a challenging problem, since the functioning of a network block depends on the activation distributions of the preceding network blocks. To address this, we developed the spatial scrambling method (see Fig 6A). This approach selectively perturbs the information for a given block, but maintains its activation distributions. As a result, this model block's features contribute less effectively to performance and the remaining, unaffected blocks will consequently dominate the model's performance (see methods for an in-depth description of spatial scrambling). Importantly, spatial scrambling led to a relative reweighting of feature contributions to performance. That is, under all circumstances, all network blocks were still affecting performance, but features that were scrambled were less informative and thus exerted less influence on the performance-gain profile. We leveraged this principle to dissect the network's performance, and determine how the features along the model hierarchy differentially contributed to performance of easy and difficult tasks. Specifically, we either increasingly added spatial scrambling from the top to the bottom of the network, thereby making the network more and more reliant on feature information contained in the early blocks or we applied it from the bottom to the top of the network, so that late network features drove performance (see Fig 6A)."

Results – Task difficulty differentially engages model features across the model hierarchy, p. 15-17

R2.S2 *The authors motivate work in terms of theories by Aston-Jones and Cohen 2005 – but it also relates to work by Mather 2016, who go into detail about how molecular mechanisms could give rise to a form of gain modulation that increases firing of active neurons, and decreases activation of less active ones (ie. results from devilbiss and waterhouse above).*

We fully agree with the reviewer that our work is also closely connected to Mather and colleagues (2016) on a conceptual level, and we point to this in our discussion:

“Crucially, this connects our model to a larger framework in which arousal regulates the efficiency of information processing (Aston-Jones & Cohen, 2005; Gelbard-Sagiv et al., 2018; Mather et al., 2016; McGinley, David, et al., 2015)”

Discussion – DCNNs with global gain reproduce key behavioral signatures linked to arousal states, p. 22

We did not cite this work as a direct precursor to our study in the introduction or methods since there are couple of key differences to the perspective promoted by Mather and colleagues: For one, we did not implement any other types of concurrent neuromodulators, such as glutamate, which however take a central role for Mather et al. (2016). Secondly, we did not distinguish between local and global release of noradrenaline and third, we did not implement any kind of direct inhibition, that could lead to the hypothesized suppression. Taken together, we deemed these differences too large to directly associate this work with the perspective offered by Mather and colleagues (2016).

References

Aston-Jones, G., & Cohen, J. D. (2005). An integrative theory of locus coeruleus-norepinephrine function: adaptive gain and optimal performance. *Annual Review of Neuroscience*, *28*, 403–450.

Beaman, C. B., Eagleman, S. L., & Dragoi, V. (2017). Sensory coding accuracy and perceptual performance are improved during the desynchronized cortical state. *Nature Communications*, *8*(1), 1308.

Berridge, C. W., & Waterhouse, B. D. (2003). The locus coeruleus-noradrenergic system: modulation of behavioral state and state-dependent cognitive processes. *Brain Research. Brain Research Reviews*, *42*(1), 33–84.

Cowley, B. R., Snyder, A. C., Acar, K., Williamson, R. C., Yu, B. M., & Smith, M. A. (2020). Slow Drift of Neural Activity as a Signature of Impulsivity in Macaque Visual and Prefrontal Cortex. *Neuron*, *108*(3), 551-567.e8.

Engel, T. A., Steinmetz, N. A., Gieselmann, M. A., Thiele, A., Moore, T., & Boahen, K. (2016). Selective modulation of cortical state during spatial attention. *Science*, *354*(6316), 1140–1144.

Faller, J., Cummings, J., Saproo, S., & Sajda, P. (2019). Regulation of arousal via online neurofeedback improves human performance in a demanding sensory-motor task. *Proceedings of the National Academy of Sciences of the United States of America*, *116*(13), 6482–6490.

Gelbard-Sagiv, H., Magidov, E., Sharon, H., Hendler, T., & Nir, Y. (2018). Noradrenaline Modulates Visual Perception and Late Visually Evoked Activity. *Current Biology: CB*, *28*(14), 2239-2249.e6.

Gutnisky, D. A., Beaman, C., Lew, S. E., & Dragoi, V. (2017). Cortical response states for enhanced sensory discrimination. *ELife*, *6*. https://doi.org/10.7554/eLife.29226

Lindsay, G. W., & Miller, K. D. (2018). How biological attention mechanisms improve task performance in a large-scale visual system model. *ELife*, *7*. https://doi.org/10.7554/eLife.38105

Luo, X., Roads, B. D., & Love, B. C. (2020). The Costs and Benefits of Goal-Directed Attention in Deep Convolutional Neural Networks. In *arXiv [cs.LG]*. arXiv. http://arxiv.org/abs/2002.02342

Mather, M., Clewett, D., Sakaki, M., & Harley, C. W. (2016). Norepinephrine ignites local hotspots of neuronal excitation: How arousal amplifies selectivity in perception and memory. *The Behavioral and Brain Sciences*, *39*, e200.

McCormick, D. A., Nestvogel, D. B., & He, B. J. (2020). Neuromodulation of Brain State and Behavior. *Annual Review of Neuroscience*, *43*, 391–415.

McGinley, M. J., David, S. V., & McCormick, D. A. (2015). Cortical Membrane Potential Signature of Optimal States for Sensory Signal Detection. *Neuron*, *87*(1), 179–192.

McGinley, M. J., Vinck, M., Reimer, J., Batista-Brito, R., Zagha, E., Cadwell, C. R., Tolias, A. S., Cardin, J. A., & McCormick, D. A. (2015). Waking State: Rapid Variations Modulate Neural and Behavioral Responses. *Neuron*, *87*(6), 1143–1161.

Nair, V., & Hinton, G. E. (2010). *Rectified Linear Units Improve Restricted Boltzmann Machines*. https://openreview.net/pdf?id=rkb15iZdZB

Neske, G. T., Nestvogel, D., Steffan, P. J., & McCormick, D. A. (2019). Distinct Waking States for Strong Evoked Responses in Primary Visual Cortex and Optimal Visual Detection Performance. *The Journal of Neuroscience: The Official Journal of the Society for Neuroscience*, *39*(50), 10044–10059.

Pfeffer, T., Ponce-Alvarez, A., Tsetsos, K., Meindertsma, T., Gahnström, C. J., van den Brink, R. L., Nolte, G., Engel, A. K., Deco, G., & Donner, T. H. (2021). Circuit mechanisms for the chemical modulation of cortex-wide network interactions and behavioral variability. *Science Advances*, *7*(29). https://doi.org/10.1126/sciadv.abf5620

Podvalny, E., Flounders, M. W., King, L. E., Holroyd, T., & He, B. J. (2019). A dual role of prestimulus spontaneous neural activity in visual object recognition. *Nature Communications*, *10*(1), 3910.

Polack, P.-O., Friedman, J., & Golshani, P. (2013). Cellular mechanisms of brain state-dependent gain modulation in visual cortex. *Nature Neuroscience*, *16*(9), 1331–1339.

Poulet, J. F. A., & Crochet, S. (2018). The Cortical States of Wakefulness. *Frontiers in Systems Neuroscience*, *12*, 64.

Reimer, J., Froudarakis, E., Cadwell, C. R., Yatsenko, D., Denfield, G. H., & Tolias, A. S. (2014). Pupil fluctuations track fast switching of cortical states during quiet wakefulness. *Neuron*, *84*(2), 355–362.

Reimer, J., McGinley, M. J., Liu, Y., Rodenkirch, C., Wang, Q., McCormick, D. A., & Tolias, A. S. (2016). Pupil fluctuations track rapid changes in adrenergic and cholinergic activity in cortex. *Nature Communications*, *7*, 13289.

Sara, S. J. (2009). The locus coeruleus and noradrenergic modulation of cognition. *Nature Reviews. Neuroscience*, *10*(3), 211–223.

Servan-Schreiber, D., Printz, H., & Cohen, J. D. (1990). A network model of catecholamine effects: gain, signal-to-noise ratio, and behavior. *Science*, *249*(4971), 892–895.

Shimaoka, D., Harris, K. D., & Carandini, M. (2018). Effects of Arousal on Mouse Sensory Cortex Depend on Modality. *Cell Reports*, *22*(12), 3160–3167.

Sörensen, L. K. A., Zambrano, D., Slagter, H. A., Bohté, S. M., & Scholte, H. S. (2020). Leveraging spiking deep neural networks to understand neural mechanisms underlying selective attention. In *bioRxiv* (p. 2020.12.15.422863). https://doi.org/10.1101/2020.12.15.422863

Stringer, C., Pachitariu, M., Steinmetz, N., Reddy, C. B., Carandini, M., & Harris, K. D. (2019). Spontaneous behaviors drive multidimensional, brainwide activity. *Science*, *364*(6437), 255.

Usher, M., Cohen, J. D., Servan-Schreiber, D., Rajkowski, J., & Aston-Jones, G. (1999). The role of locus coeruleus in the regulation of cognitive performance. *Science*, *283*(5401), 549–554.

Vinck, M., Batista-Brito, R., Knoblich, U., & Cardin, J. A. (2015). Arousal and locomotion make distinct contributions to cortical activity patterns and visual encoding. *Neuron*, *86*(3), 740–754.

Waschke, L., Tune, S., & Obleser, J. (2019). Local cortical desynchronization and pupil-linked arousal differentially shape brain states for optimal sensory performance. *ELife*, *8*. https://doi.org/10.7554/eLife.51501

Wischnewski, M., & Peelen, M. V. (2021). Causal Evidence for a Double Dissociation between Object- and Scene-Selective Regions of Visual Cortex: A Preregistered TMS Replication Study. *The Journal of Neuroscience: The Official Journal of the Society for Neuroscience*, *41*(4), 751–756.
