# Supplementary material for: Arousal state affects perceptual decision-making by modulating hierarchical sensory processing in a large-scale visual system model
Source: PLoS Comput Biol. 2022 Apr 4;18(4):e1009976. doi: 10.1371/journal.pcbi.1009976 (PMC9009767; doi:10.1371/journal.pcbi.1009976)
Supplement: Attachment — Submitted filename: ResponseToReviewers.pdf [file pcbi.1009976.s008.pdf]

Amsterdam, February 24, 2022

Dear Dr. Isik, dear Dr. Serre,

We hereby submit our revised manuscript "Arousal state affects perceptual decision-making by modulating hierarchical sensory processing in a large-scale visual system model" for publication in PLOS Computational Biology.

We were pleased to hear that we addressed the majority of the reviewer's concerns with our last version of our manuscript. Below we have addressed the two remaining comments by reviewer 2, with the relevant parts of the original comments in italics, and our responses below that. We hope that the manuscript, which was revised accordingly, is now suitable for publication.

Thank you for your time and consideration.

Sincerely,
On behalf of all authors,
Lynn Sörensen

Responses to reviewer 2

R2.1 *While the ReLu instantiation of the model rules out one possible mechanism through which the observed effects arise, it doesn't answer the larger question of how the effects DO arise in the current setup. I can appreciate that the incremental scrambling approach is intended to get at this question, but at the end of the day I don't find the results of that experiment to provide a satisfying explanation of how the effects come about. To me, a satisfying explanation would require examination of what sorts of information are differentially propagated from layer to layer as you change gain, which almost certainly would require understanding how gain changes in one layer push neural inputs in the next layer towards or away from nonlinearities in the activation function. I think this would be addressable in toy problems. That said, I understand that this may be a question for future work.*

We agree with the reviewer that our current scrambling results are only a starting point for understanding the effects at play here. As the reviewer suggests, studying the interaction between a global gain mechanism and hierarchical processing in a smaller-scale model could be an important next step for future work. We have added a few sentences to the discussion to direct to this avenue suggested by the reviewer:

"Future studies should also examine how specifically information is differentially propagated throughout the hierarchy as result of global gain changes, for example, by studying the interaction between global gain changes and computational circuits, such as ResNet blocks, in smaller-scale models. They should also extend our findings to other architectures to ensure that our findings generalize beyond ResNet architectures. We hope that our model will serve as an important steppingstone for experimentally and theoretically investigating how global state changes, such as arousal, affect sensory processing and perception."

Discussion, p. 21

R2.2 *The long-timescale changes in arousal (as measured with pupil dilation) reported in Vinck, that the authors note relating to higher firing rates in visual regions, are unlikely to be related to norepinephrine signaling, given other work showing that relationship between NE and pupil dilation only holds for high frequency fluctuations (Reimer 2016; Joshi 2016). That said, to the best of my knowledge, the behavioral phenomena that are modeled in the paper have not been explicitly linked to the NE system either. So I would just suggest that the authors use a bit more caution in linking behavioral phenomena to underlying neural systems.*

We appreciate the reviewer's remark and we have adjusted the wording throughout the manuscript (italics in quotes below).

“It is *commonly proposed* that the noradrenergic locus coeruleus system regulates these effects on performance through a widespread release of noradrenaline resulting in changes of cortical gain.”

Abstract, p. 1

“These findings critically inform current debate as to how arousal *may* impact perceptual decision-making.”

Introduction, p. 5

“Arousal has been *linked* to increases in firing rates in early visual cortex in response to sensory stimulation, that is, to increases in sensory gain, e.g. [28]). These increases in gain *were suggested* to be a result of a global release of noradrenaline *(in conjunction with other neuromodulators [52])*, making them spatially unspecific [4,14,16,48,52].”

Methods – Global gain mechanism, p. 26
